# Epidemiological and Pathogenic Relationship between Sleep Apnea and Ischemic Heart Disease

**DOI:** 10.1155/2013/405827

**Published:** 2013-06-18

**Authors:** Carlos Carpio, Rodolfo Álvarez-Sala, Francisco García-Río

**Affiliations:** Servicio de Neumología, Hospital Universitario La Paz, Instituto de Investigación Sanitaria del Hospital Universitario La Paz (IdiPAZ), 28046 Madrid, Spain

## Abstract

Obstructive sleep apnea is recognized as having high prevalence and causing remarkable cardiovascular risk. Coronary artery disease has been associated with obstructive sleep apnea in many reports. The pathophysiology of coronary artery disease in obstructive sleep apnea patients probably includes the activation of multiple mechanisms, as the sympathetic activity, endothelial dysfunction, atherosclerosis, and systemic hypertension. Moreover, chronic intermittent hypoxia and oxidative stress have an important role in the pathogenesis of coronary disease and are also fundamental to the development of atherosclerosis and other comorbidities present in coronary artery diseases such as lipid metabolic disorders. Interestingly, the prognosis of patients with coronary artery disease has been associated with obstructive sleep apnea and the severity of sleep disordered breathing may have a direct relationship with the morbidity and mortality of patients with coronary diseases. Nevertheless, treatment with CPAP may have important effects, and recent reports have described the benefits of obstructive sleep apnea treatment on the recurrence of acute heart ischaemic events in patients with coronary artery disease.

## 1. Introduction

Obstructive sleep apnea (OSA) is a common medical condition characterized by abnormal collapse of the pharyngeal airway during sleep, causing repetitive arousals, and drops in the oxygen saturation. It is highly prevalent in the general population [1] and it acts as an independent risk factor for hypertension (HT) [2, 3]. In addition, several studies have suggested that OSA is associated with other cardiovascular diseases such as heart failure [4], arrhythmias [5], pulmonary hypertension [6], and coronary artery disease (CAD).

Although mortality from CAD has fallen since 1975, it is still a major cause of death and disability in developed countries. The high prevalence of cardiovascular risk factors in the general population (diabetes mellitus, cigarette smoking, obesity, hypertension, and lack of regular physical activity) facilitates the development of atherosclerosis in coronary arteries and, subsequently, the presence of CAD. Although clinical guidelines for the management of CAD still do not consider OSA as a specific risk factor, results from the Sleep Heart Health Study have shown that OSA may increase the risk of CAD in middle-aged men with an apnea-hypopnea index (AHI) ≥ 30 h^−1^ [7]. 

## 2. Association between Obstructive Sleep Apnea and Coronary Artery Disease

 Several cross-sectional and epidemiological studies have evaluated the association between OSA and CAD. In a case-control study, Mooe et al. [8] selected 102 women with CAD and 50 age-matched controls and reported that CAD patients had a higher prevalence of sleep breathing disorders than the control group (AHI ≥ 5 h^−1^: 54% versus 20%, *P* < 0.0001; AHI ≥ 10 h^−1^: 31% versus 18%, *P* < 0.05, resp.). These findings were also described in men, where authors observed that 37% of patients with CAD had an AHI ≥ 10 h^−1^ [9]. Despite their interesting findings, these studies had several limitations. For instance, important cardiovascular risk factors, such as cholesterol levels, were not included in the analysis. Also, in the manual score of the sleep study, authors did not discriminate between central and obstructive apneas. And finally, a questionnaire was only used about coronary symptoms to exclude CAD in the control subjects. A similar association had been found in a population cross-sectional study. Shahar et al. [10], analyzed the association between OSA and self-reported cardiovascular disease in 6.424 individuals who underwent overnight polysomnography from the Sleep Heart Health Study. They showed that subjects with the highest quartile of AHI (AHI > 11 events h^−1^) had an adjusted ratio of 1.27 for self-reported CAD. Recently, Gottlieb et al. [7] have found a significant association between severe OSA and coronary heart disease in middle-aged men from the Sleep Heart Health Study, and they reported a significant association between severe OSA and coronary heart disease in middle-aged men.

 There is some evidence suggesting that the prevalence and the severity of OSA are modified along the CAD evolution. Moruzzi et al. [11] performed overnight polysomnographic studies in three groups of CAD patients: immediately after an acute myocardial infarction (AMI) (group 1), after clinical stabilization of unstable angina (group 2), and with stable angina (group 3). They observed a significantly higher AHI in groups 1 and 2 compared with group 3 (11.1 ± 19.4 h^−1^, 14.7 ± 5.20 h^−1^, and 2.8 ± 6.4 h^−1^, resp.; *P* < 0.01). However, studies from Mooe et al. [8] and Moruzzi et al. [11] did not adjust properly for confounding factors. So Peker et al. [12] performed a case-control study adjusted for several cardiovascular risk factors that included hypertension, hypercholesterolemia, diabetes, and smoking, and compared the results of sleep recording studies of 62 patients admitted in the coronary care unit due to acute CAD and 62-age-, sex-, and BMI-matched control subjects without history or signs of heart disease. OSA (AHI ≥ 10 h^−1^) was present in 19 CAD patients but only in 8 control subjects (*P* = 0.017). In the multiple logistic regression analysis, current smoking (odds ratio [OR] 9.8, 95% CI 2.6–36.5), diabetes (OR 4.2, 95% CI 1.1–17.1), and OSA (OR 3.1, 95% CI 1.2–8.3) were independently associated with coronary disease. However, this study also had several limitations as the high prevalence of hypercholesterolemia in both groups (>80% of patients), the sleep diagnostic procedure used, and the substantial delay between the cardiac event and the sleep study (4–21 months). Supporting the relationship between OSA and CAD, Lee et al. [13] and Nakashima et al. [14] have also found similar results and they have observed a moderate-severe OSAS (AHI ≥ 15 h^−1^) in 43% and 65.7% of subjects with CAD, respectively. Despite the high prevalence of OSA reported in patients with CAD, it is frequently underdiagnosed. One study demonstrated that OSA was initially suspected in only 12% of patients admitted with myocardial infarction (MI) whereas, after an overnight polysomnography, 70% of patients presented an AHI ≥ 5 h^−1^ and 41% an AHI ≥ 15 h^−1^ [15].

There are several explanations for the difference in the OSA prevalence among the aforementioned studies. The definition of the ideal timing for OSA screening after an acute cardiovascular event remains unresolved. Skinner et al. [16] performed two overnight sleep studies in patients with CAD (MI, unstable angina or congestive heart failure): at the time of acute presentation in the Coronary Care Unit and at least six weeks after hospital discharge. They identified an AHI ≥ 15 h^−1^ in 13 of 26 patients (50%) in the first sleep study but in only 5 of 18 patients (28%) during the second study. In fact, it has been reported that the AHI obtained by overnight polysomnography significantly decreases in the CAD chronic phase (day 14) with respect to the acute phase (days 3–5) (6.97 ± 5.67 versus 13.26 ± 11.30, resp.) [17]. Moreover, it seems that alterations in sleep architecture in CAD subjects tend to decrease over time. In accordance with this line, Schiza et al. [18] performed a full-night polysomnography in 22 patients with acute coronary syndrome (ACS) within 3 days of the first episode and 1 and 6 months later. They found a progressive increase in the total sleep time, sleep efficiency, slow wave sleep, and rapid eye movement (REM) sleep. Despite this, an early identification of OSA in subjects admitted with ACS does not affect the odds of hospital readmission in the next six months [19]. Possible reasons that explain why patients in the acute phase of CAD have higher OSA diagnosis rate have been reported and they are related to the supine position of patients in the Coronary Care Unit, the fragmented sleep with reduced rapid eye movement stage in the first night and finally, the sleep breathing disorders that produce coronary ischemic diseases by itself (central apneas). 

Not all studies have an adequate assessment of conditions that may increase the prevalence of sleep-disordered breathing, such as sedation or narcotics use, COPD, alcoholism, level of consciousness, and stroke. Additionally, the sleep diagnostic tests differ in several studies, and although attended polysomnography is considered standard practice in patients with related medical comorbid conditions [13, 17], many studies have used nonattended sleep studies [14, 15]. Moreover, definitions of respiratory events have not been homogeneous. For example, Lee et al. [13], consider a 3% decrease in oxygen saturation to define hypopneas, while Nakashima et al. [14] considered them when the drop of oxygen saturation was equal or greater than 4%. 

## 3. Mechanisms of Coronary Artery Disease in Obstructive Sleep Apnea

Systemic hypertension is a risk factor for the development of CAD and guidelines for the management of hypertension consider that OSA contributes directly to the pathogenesis of hypertension [3, 20]. OSA is characterized by recurrent episodes of upper airway collapse during sleep, which is accompanied by cycles of hypoxia-reoxygenation leading chronic intermittent hypoxia (CIH). In animal models, it has been shown that intermittent hypoxia could be associated with the development of hypertension [21]. Moreover, CIH may affect the plasma renin-angiotensin activity, the production of endothelin, and the function of peripheral chemoreceptors, increasing the sympathetic activity [22]. OSA patients have higher plasma and urinary catecholamine levels than control subjects [23–25] and the disbalance between sympathetic and parasympathetic systems may increase systemic vascular resistance and blood pressure [26]. Furthermore, evidence from animal models supports that CIH contributes to vascular remodeling [27] and Drager et al. [28] reported that an increased carotid intima-media thickness is associated to sympathetic activity and atherosclerosis.

OSA patients present systemic inflammation related to endothelial dysfunction. CIH has been associated with endothelial dysfunction independently of other risk factors for atherosclerosis such as obesity, dyslipidemia, diabetes, or smoking [29]. Moreover, patients with OSA have an increased number of apoptotic endothelial cells and fewer circulating progenitor cells [30, 31]. Finally, endothelial dysfunction has been also associated with a reduced availability of nitric oxide in subjects with OSA [29, 32].

The third mechanism involved in the pathogenesis of CAD in patients with OSA is due to the development of atherosclerotic plaques through metabolic, oxidative, and inflammatory pathways. Recurrent episodes of hypoxia are associated with adipose tissue dysfunction and production of different adipokines. Increased leptin levels observed in OSA are related to endothelial dysfunction, production of cytokines, platelet aggregation, and oxidative stress [33, 34]. Moreover, obstructive apneas downregulate adiponectin levels, which are closely associated with endothelial dysfunction and atherosclerosis [35]. Furthermore, dyslipidemia is present in many OSA subjects and it is characterized by an increased synthesis and secretion of very LDL-cholesterol and triglycerides and a reduced secretion of  HDL-cholesterol, promoting atherogenesis [36, 37].

**Figure 1:**
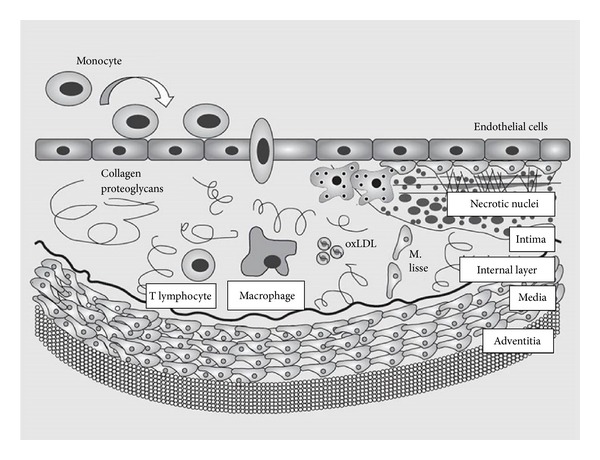
Development of atherosclerotic plaques in obstructive sleep apnea patients, modified from Nácher et al. [39].

Production of some proinflammatory mediators such as TNF-*α*, IL-1, IL-8, and adhesion molecules are promoted by lipid peroxidation and endothelial dysfunction, resulting in an environment of systemic inflammation. This facilitates the recruitment and accumulation of macrophages and fat cells that further activates lipid peroxidation and promotes endothelial cell damage and atherosclerosis [38, 39]. C-reactive protein (CRP), another inflammatory biomarker, has been associated with ACS. Higher CRP levels have been reported in OSA patients, but it is difficult to tease out the independent contribution of obesity on the CRP levels [40, 41] (Figure 1).

This inflammatory process associated to CIH, endothelial dysfunction and atherosclerosis, is induced and regulated by several transcription factors, such as kappa-B nuclear factor (NF-kB) and hypoxia inducible factor (HIF)-1*α* [42, 43]. They have a key role in the regulation of the innate immunity and participate actively in inflammatory pathway. Also, in animal models, they have been implicated in hypertension and in components of the metabolic syndrome. However, the potential role of these and other transcription factors in the pathogenesis of CAD should be further investigated in the future, and thereby, combining with individual gene analysis and personalized medicine, may provide new treatment strategies for cardiovascular protection.

## 4. Influence of Sleep-Disordered Breathing on the CAD Prognosis

There has been growing evidence associating OSA with prognosis of CAD, both in stable and unstable patients. Peker et al. [44] observed, during a follow-up period of 5 years, the cardiovascular mortality of 62 consecutive CAD stable patients with and without OSA. They found 6 cardiovascular deaths in OSA patients (37.5%) and 4 in the non-OSA group (9.3%) (*P* = 0.018). Additionally, in the Cox multiple conditional regression model, respiratory disturbance index (RDI) was found to be an independent predictor of cardiovascular mortality (hazard ratio [HR] = 1.13; 95% CI 1.05–1.21, *P* < 0.001). Main limitations of this study concern aspects that could affect the prevalence of OSA, as the type of sleep studies and the timing of OSA screening (4–21 months after admission).

Moreover, OSA appeared to affect clinical and angiographic outcomes after percutaneous coronary intervention (PCI) in patients with ACS. In fact, Yumino et al. [45] performed a sleep study in 89 patients with ACS and, after a follow-up mean period of 227 days, they observed a higher incidence of major adverse cardiac events (cardiac death, reinfarction, and target vessel revascularization) in patients with OSA (AHI > 10 h^−1^) (23.5% versus 5.3%, *P* = 0.022). Furthermore, OSA was an independent predictor for major adverse cardiac events (HR 11.62, 95% CI 2.17–62.24; *P* = 0.004) and of subsequent angiographic binary restenosis (HR 7.69, 95% CI 1.74–34.05; *P* = 0.007). Evidence linking sleep-disordered breathing to increased mortality and cardiovascular morbidity has been conflicting and inconclusive in patients with established CAD. On one hand, Mooe et al. [46] in a prospective cohort of 408 patients with chronic CAD followed during a median period of 5.1 years found that there was a 70% and a 62% relative increase in the primary end point (composite of death, cerebrovascular events, and myocardial infarction) in patients with a desaturation index (DI) ≥ 5 h^−1^ and an AHI ≥ 10 h^−1^, respectively. On the other hand, Hagenah et al. [47] evaluated the prognostic influence of OSA in 50 patients with stable CAD. After 10 years of follow-up, they observed that OSA did not increase the risk of mortality and cardiovascular complications. However, both groups were not fully comparable, having non-OSA patients with a tendency to more severe coronary lesions.

Myocardial tissue perfusion after primary PCI plays a pivotal role in recovery of left ventricular function and patient prognosis in the clinical setting of ACS [48]. Nakashima et al. [49] used systolic retrograde flow (SRF) and ST-segment resolution (STR) <50% to measure myocardial tissue perfusion immediately after the PCI in 100 patients with ACS. They performed overnight polysomnography at 14 days of admission in all patients and they found in the multiple logistic regression analysis that OSA induced more severe microvascular injury related to ischemia-reperfusion (SRF: OR = 4.46, *P* = 0.044; STR: OR = 3.79, *P* = 0.010). However, Lee et al. [13] did not find similar results. To measure impaired microvascular perfusion after primary PCI, these authors used ST-segment resolution of <70%, myocardial blush grade 0 or 1, or a corrected Thrombolysis in Myocardial Infarction (TIMI) frame count >28. A sleep study was performed and completed in 105 patients and OSA was not found associated with impaired microvascular perfusion after primary PCI. These differences could be related to the different technique used to measure myocardial perfusion in both studies. Moreover, differences in the timing of sleep studies (Lee et al. [13] performed the sleep study 14 to 21 days after hospital admission, whereas Nakashima et al. [49] did it 2 to 5 days after hospital admission) could affect the prevalence of OSA in CAD subjects.

## 5. CPAP Effect on the Prognosis of Ischemic Cardiac Disease

Several observational studies have reported that CPAP may reduce cardiovascular mortality in OSA patients. One study showed that the incidence of fatal and nonfatal cardiovascular events during 10 years of follow-up was higher in untreated severe OSA patients than in patients treated with CPAP or in healthy subjects [50]. Furthermore, Buchner et al. [51] reported that treatment of OSA decreases the risk of fatal and nonfatal cardiovascular events (myocardial infarction, stroke, and ACS requiring revascularization procedures) in patients with mild-moderate OSA (HR 0.36, 95% CI 0.21–0.62, *P* = 0.001) after 72-month follow-up. However, in this study, the mean duration of follow-up was significantly different between untreated and treated patients (50.0 ± 49.4 versus 77.0 ± 55.0 months; *P* = 0.001). Moreover, both Marín et al. and Buchner el al. studies did not have a controlled, randomized design, so their findings could not be used to make causal inferences.

Few studies have assessed the effects of CPAP on the morbidity and mortality of CAD patients with OSA, but some recent reports confirm the possible positive impact of OSA treatment on the prognosis of CAD. An early report compared the occurrence of a composite endpoint (cardiovascular death, ACS, hospitalization for heart failure, or need for coronary revascularization) between OSA patient who accepted (CPAP = 11 patients, upper airway surgery = 4 patients) and declined OSA treatment (29 patients). At the end of follow-up (86.5 ± 39 months), the endpoint was reached in 6 (24%) and 17 (58%) patients with and without OSA treatment, respectively (*P* < 0.01). Moreover, OSA treatment reduced in 62% the risk of occurrence of the composite endpoint (HR 0.24; 95% CI: 0.09–0.62; *P* < 0.01) [52]. Cassar et al. [53] added further information to the study of Milleron et al. [52]. They designed a retrospective cohort study of 371 patients diagnosed with OSA (AHI ≥ 15 h^−1^) who subsequently underwent a PCI and evaluated cardiac death, general mortality, major adverse cardiac events (MACE) (severe angina, myocardial infarction, PCI, coronary artery bypass grafting, or death), and major adverse cardiac or cerebrovascular events (MACCE). They observed that patients treated for OSA had a statistically significant decreased number of cardiac deaths on 5-year follow-up when compared with untreated OSA patients (3% versus 10%, *P* = 0.027), as well as a trend toward decreased all-cause mortality (11% versus 17%, *P* = 0.058). However, there was no difference in the number of MACE or MACCE between the 2 groups.

Two recent studies performed in Spain have analyzed the efficacy of CPAP treatment in cardiovascular diseases. Barbé et al. [54], in a multicenter, parallel, randomized controlled trial, assigned in a 1 : 1 ratio to receive CPAP treatment or no active intervention in 723 nonsleepy OSA patients. They compared the incidence of either systemic hypertension or cardiovascular events (nonfatal myocardial infarction, nonfatal stroke, transient ischemic attack, hospitalization for unstable angina or arrhythmia, heart failure, or cardiovascular death). At the end of follow-up (4 years), there were no differences in the hypertension or cardiovascular event incidence density rate (IDR) between CPAP and control groups. However, in the post hoc analysis, patients with CPAP adherence of ≥4 hours per night had a lower incidence of hypertension or cardiovascular events than control group (IDR: 0.69, 95% IC 0.50–0.94, *P* = 0.02).

**Figure 2:**
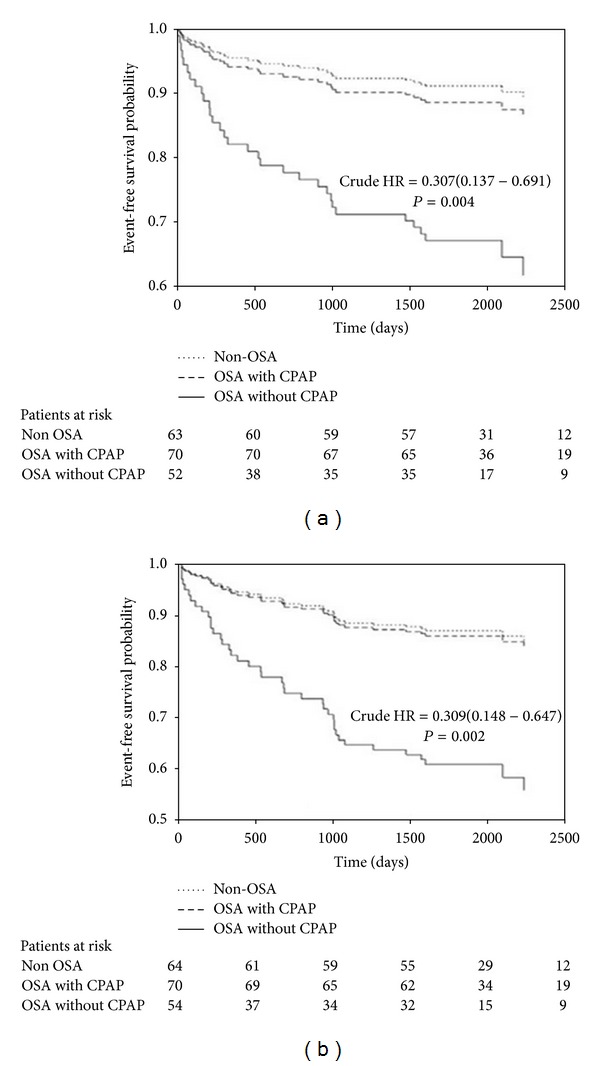
Time until first recurrent myocardial infarction (a) or revascularization procedure (b) in the three groups of MI patients. Crude hazard ratio (HR) of treated versus untreated OSA is presented, reproduced from Garcia-Rio et al. [55].

Recently, we have analyzed the evolution of 192 patients diagnosed with MI and 96 control subjects after a follow-up period of 6.5 years. OSA was an independent predictor of AMI (OR 4.9, IC 95% 2.9–8.3, *P* = 0.017), with directly proportional relationship. Furthermore, we have observed that treated OSA patients had a lower risk of recurrent AMI (HR 0.16, IC 95% 0.03–0.76, *P* = 0.021) and revascularization (HR 0.15, IC 95% 0.03–0.79, *P* = 0.025) than untreated OSA patients, but a similar risk to non-OSA patients [55] (Figure 2). 

## 6. Conclusions

OSA is a common condition that is associated with several cardiovascular complications such as CAD. The CAD pathogenesis in OSA is complex and probably related to increased sympathetic activity, endothelial dysfunction, and atherosclerosis. In addition, abnormal lipid metabolism related to OSA could also participate in the pathogenesis of CAD. Several studies have reported an association between OSA and CAD in stable and unstable patients. Furthermore, the prognosis of subjects with coronary disease could be affected by the presence and the severity of OSA. Finally, there is evidence that OSA treatment with CPAP reduces the mortality and morbidity of CAD patients, but there is still a need for long-term data to confirm the benefit of CPAP treatment.
